# Attrition and Adherence in a Web-Based Distress Management Program for Implantable Cardioverter Defibrillator Patients (WEBCARE): Randomized Controlled Trial

**DOI:** 10.2196/jmir.2809

**Published:** 2014-02-28

**Authors:** Mirela Habibović, Pim Cuijpers, Marco Alings, Pepijn van der Voort, Dominic Theuns, Leon Bouwels, Jean-Paul Herrman, Suzanne Valk, Susanne Pedersen

**Affiliations:** ^1^Center of Research on Psychology in Somatic DiseasesDepartment of Medical and Clinical PsychologyTilburg UniversityTilburgNetherlands; ^2^Department of Clinical PsychologyVrije UniversityAmsterdamNetherlands; ^3^Department of CardiologyAmphia HospitalBredaNetherlands; ^4^Department of CardiologyCatharina HospitalEindhovenNetherlands; ^5^Department of CardiologyErasmus Medical CenterRotterdamNetherlands; ^6^Department of CardiologyCanisius-Wilhelmina HospitalNijmegenNetherlands; ^7^Department of CardiologyOnze Lieve Vrouwe GasthuisAmsterdamNetherlands; ^8^Department of CardiologyVlietland HospitalSchiedamNetherlands; ^9^Department of PsychologyUniversity of Southern DenmarkOdense MDenmark; ^10^Department of CardiologyOdense University HospitalOdenseDenmark

**Keywords:** implantable cardioverter defibrillator, Web-based interventions, adherence, dropout, attrition

## Abstract

**Background:**

WEB-Based Distress Management Program for Implantable CARdioverter defibrillator Patients (WEBCARE) is a Web-based randomized controlled trial, designed to improve psychological well-being in patients with an implantable cardioverter defibrillator (ICD). As in other Web-based trials, we encountered problems with attrition and adherence.

**Objective:**

In the current study, we focus on the patient characteristics, reasons, and motivation of (1) completers, (2) those who quit the intervention, and (3) those who quit the intervention and the study in the treatment arm of WEBCARE.

**Methods:**

Consecutive first-time ICD patients from six Dutch referral hospitals were approached for participation. After signing consent and filling in baseline measures, patients were randomized to either the WEBCARE group or the Usual Care group.

**Results:**

The treatment arm of WEBCARE contained 146 patients. Of these 146, 34 (23.3%) completed the treatment, 88 (60.3%) dropped out of treatment but completed follow-up, and 24 (16.4%) dropped out of treatment and study. Results show no systematic differences in baseline demographic, clinical, or psychological characteristics between groups. A gradual increase in dropout was observed with 83.5% (122/146) completing the first lesson, while only 23.3% (34/146) eventually completed the whole treatment. Reasons most often given by patients for dropout were technical problems with the computer, time constraints, feeling fine, and not needing additional support.

**Conclusions:**

Current findings underline the importance of focusing on adherence and dropout, as this remains a significant problem in behavioral Web-based trials. Examining possibilities to address barriers indicated by patients might enhance treatment engagement and improve patient outcomes.

**Trial Registration:**

Clinicaltrials.gov: NCT00895700; http://www.clinicaltrials.gov/ct2/show/NCT00895700 (Archived by WebCite at http://www.webcitation.org/6NCop6Htz).

## Introduction

The implantable cardioverter defibrillator (ICD) is a cardiac device that is implanted with leads in and on the heart in patients for the primary and secondary prevention of sudden cardiac death due to life-threatening ventricular tachyarrhythmias [1,2]. In case of a ventricular arrhythmia, the ICD paces or delivers a low- or high-voltage electric shock to the heart muscle to terminate the arrhythmia and return the heart to a normal rhythm [1,2]. The experience of the ICD shock is often described by patients as “getting kicked in the chest by a big horse”, although patients’ experiences vary considerably [3,4].

A subgroup of ICD patients experiences psychological distress after ICD implantation, such as anxiety, depression, post-traumatic stress, and impaired quality of life [5,6]. However, distress in patients may not necessarily be attributed to the device and ICD therapy, but also to the underlying disease [7,8] and patients’ pre-implantation psychological profile [9]. The importance of monitoring ICD patients for psychological distress is increasingly being recognized [10], likely due to risk for morbidity and mortality associated with patient distress [11,12] and due to preliminary evidence suggesting that distress is still undertreated in this population [13]. One possible explanation for this is the lack of well-designed, validated, randomized controlled trials (RCTs), leaving us with insufficient knowledge for the establishment of a sufficient evidence base to inform clinical practice [14]. Results of previous trials have been promising with respect to reducing distress, but the majority of these trials had a high dropout rate, jeopardizing the external validity of these studies [15,16].

In order to make psychological treatment for ICD patients more patient-tailored, which may reduce dropout, the use of online Web-based interventions has been advocated [15,17]. There is evidence showing that Web-based interventions are as effective as face-to-face interventions [18,19], and might be able to overcome barriers such as travel burden, time constraints, and reluctance to seek help, and thus reach underserved groups of patients [20,21]. However, adherence and attrition also remain major challenges in these trials [22,23]. To date, the reasons for dropout are not well understood and deserve attention in their own right, in order to increase the success and applicability of results of future Web-based intervention trials.

In the current study, we will describe the attrition and adherence issues that we encountered during the “WEB-based distress management program for implantable CARdioverter dEfibrillator” patients (WEBCARE) trial (NCT00895700). The trial design paper was published previously [24]. In brief, the aim of WEBCARE was to reduce anxiety and depression and improve the quality of life of patients with a first-time ICD implant through a 12-week Web-based intervention (called “Leef met je hart” or “Live with your heart”), using cognitive behavioral therapy as the mainstay of treatment combined with psycho-education related to the ICD and relaxation techniques. Specific objectives were to (1) examine whether (a) completers, (b) patients who dropped out of treatment but remained in the study (filled in follow-up questionnaires), and (c) patients who dropped out of the treatment and the study, differ systematically on baseline demographic, clinical, and psychological characteristics, and (2) present descriptive data on patients’ reasons for dropping out.

## Methods

### Patient Population

Consecutively implanted ICD patients from six hospitals in the Netherlands (ie, Erasmus Medical Centre, Rotterdam; Amphia Hospital, Breda; Catharina Hospital, Eindhoven; Onze Lieve Vrouwe Gasthuis, Amsterdam; Canisius Wilhelmina Hospital, Nijmegen; Vlietland Hospital, Schiedam) were approached for study participation between April 2010 and February 2013. Patients were eligible for participation if they fulfilled the following inclusion criteria: first-time ICD implant, age 18-75 years, proficient in the Dutch language, and with Internet access and a sufficient level of Internet skills. Exclusion criteria were the following: life expectancy less than 1 year, history of psychiatric illness other than affective/anxiety disorders, or on the waiting list for heart transplantation.

### Data Collection

Patients were approached by the ICD nurse or ICD technician prior to or briefly after ICD implantation. They were informed both verbally and in writing about the study. If the patient met the inclusion criteria and was willing to participate, informed consent was signed. Patients who could not decide at that time were approached again after ICD implant while still hospitalized. Prior to discharge from the hospital, consented patients were provided with the first set of questionnaires (baseline) and their medical records were accessed for information on their demographic and clinical variables. After completing the questionnaires, patients returned them in a self-addressed and pre-stamped envelope to Tilburg University, Netherlands, which served as the core lab for WEBCARE. If the questionnaires were not returned within two weeks, patients received up to 3 reminder phone calls. Patients who did not want to participate but who were willing to give access to information from their medical records also signed an informed consent form. The study was approved by the Medical Ethics Committee of all participating centers and was conducted in accordance with the Declaration of Helsinki. All patients provided written informed consent.

### Design and Randomization

After receiving the baseline questionnaires and signed informed consent, participants were randomly assigned to either of two conditions: (1) the WEBCARE (WC) group, receiving questionnaires at baseline, 3 months, 6 months, and 12 months by mail, and getting access to the Web-based intervention for a time period of 12 weeks to complete 6 modules online [24] in addition to usual care, or (2) the Usual Care (UC) group, receiving only questionnaires by mail at all time points and usual care.

Patients were randomized using block randomization by computer, randomizing 20 patients per hospital, at each time point. Randomization lists were generated by an independent, blinded statistician and sealed by a research assistant. For the current analyses, we will only focus on patients who were randomized to the WC group.

### Descriptive Data

Patients who signed the informed consent form but who decided to quit the intervention and/or the study prematurely were contacted by telephone 12 weeks after randomization and asked why they had decided to quit. This time interval was chosen in order to not interfere with possible intervention effects (patients were allowed to work at their own pace, some chose to finish the intervention within the first two weeks, while others decided to do the 6 lessons within the last two weeks. For that reason, it was clear at 12 weeks who had quit or finished the intervention). Hence, patients were contacted at the time that they should have received their 3-month follow-up and finished the 6-module online course.

### Intervention: “Live with your Heart” (Leef met je Hart)

The intervention was based on the previously developed Web-based treatment “Alles Onder Controle” *(*Everything Under Control*)* [25] and was for the purpose of the WEBCARE trial adapted for ICD patients. The Alles Onder Controle treatment was developed for the healthy depressed population and has proven to be effective in reducing distress [25,26]. The Web-based course for ICD patients is a 12-week intervention of 6 online lessons addressing distress based on the cognitive behavioral model (problem-solving treatment). The first lesson focused on psycho-education with respect to living with an ICD (eg, what are “normal” adaptation problems post ICD implantation). In the second lesson, patients received homework assignments and were provided with therapist feedback (feedback was provided by master’s-level psychologists and was intended as minimal guidance to help patients get through the lessons—encouraging patients to continue with the lessons and giving guidance on how to address their problems according to problem-solving theory). In addition, patients received a relaxation training CD, which they were allowed to use throughout the intervention.

Patients were allowed to work at their own time and pace; however, if a lesson was not finished within two weeks, a reminder email was sent, with up to 3 reminders per lesson. Patients could proceed to the next lesson only when the previous one was finished and the homework assignment was sent to the therapist. If patients did not log in within the first two weeks, a reminder email was sent. Twelve weeks after receiving the log-in information, patients’ accounts were automatically closed.

### Measures

#### Demographic and Clinical Measures

Information on demographic variables (ie, age, gender, working status, marital status, education level) was collected through purpose-designed questions in the questionnaires, while information on clinical variables (ie, left ventricular ejection fraction [LVEF], QRS-width [electrocardiogram reading], New York Heart Association functional class [NYHA-class], presence of heart failure, use of cardiac and psychotropic medication) were extracted from patients’ medical records at the time of implantation by the implanting electrophysiologist or research nurses at the participating centers. The Charlson Comorbidity Index [27] was calculated based on self-report data and information from patients’ medical records.

#### Anxiety

The Generalized Anxiety Disorder scale (GAD-7) was used to assess anxiety [28]. The GAD-7 is a 7-item self-report questionnaire assessing anxiety symptoms in the past two weeks (eg, “Feeling nervous, anxious, or on the edge”). The GAD-7 is a reliable measure, with a Cronbach alpha of .92 and an intraclass correlation of .83 [28]. The 7 items are rated on a 4-point Likert scale from 0 (not at all) to 3 (almost every day) (score range 0-21), with a higher score indicating increased anxiety symptoms.

#### Depression

The Patient Health Questionnaire (PHQ-9) is a 9-item self-report measure of depression (eg, “Having little interest or pleasure in doing things”) that taps into the 9 diagnostic criteria for DSM-IV depressive disorder [29]. The PHQ-9 can establish provisional depressive disorder diagnoses as well as grade depressive symptom severity. Items are evaluated on a 4-point Likert scale from 0 (not at all) to 3 (almost every day) (score range 0-27), with a higher score indicating more depressive symptoms [29]. The PHQ-9 has excellent reliability with a Cronbach alpha of .91 and good validity, and has previously been used in cardiac patients [30].

#### Type D (Distressed) Personality

Type D personality was assessed with the 14-item Type D scale (DS14) [31], which consists of two 7-item subscales measuring Negative Affectivity (eg, “I often feel unhappy”) and Social Inhibition (eg, “I am a “closed” kind of person”) [32]. Items are answered on a 5-point Likert scale ranging from 0 (false) to 4 (true), with total scores on both subscales ranging from 0 to 28. A standardized cut-off ≥10 on both subscales defines individuals with a Type D personality, as Item Response Theory has indicated this to be the most optimal cut-off [31,32]. Both subscales are internally consistent, with a Cronbach alpha of .88 for Negative Affectivity and .86 for Social Inhibition. The test-retest reliability for the two subscales over a 3-month period were *r*=0.72 and 0.82, respectively [33].

#### Optimism and Pessimism

The Life Orientation Test (LOT) was used to assess optimism and pessimism [34]. Optimism was measured using a sum score of items 1, 4, 5, and 11; while pessimism was assessed with the sum score of items 3, 8, 9, and 12. Items are answered on a 5-point Likert scale from 0 (very much disagree) to 4 (very much agree)*.* The total score for the optimism and pessimism subscales ranges between 0 and 16, with a higher score indicating higher levels of the respective trait [34].

### Statistical Analyses

Continuous variables were compared using the Student’s *t* test, while discrete variables were compared using the chi-square test. Data are represented as percentages for nominal variables and mean (SDs) for continuous variables. To compare groups on psychological variables, ANOVAs (analysis of variance) were performed. If group differences were observed, the Tukey-Kramer post-hoc test for unequal group sizes was used to identify which groups differed significantly. Descriptive data were coded and analyzed using “frequencies”. A *P*<.05 indicated statistical significance. All tests were two-tailed. Data were analyzed using SPSS Statistics 19.0 for Windows.

## Results

### Patient Characteristics

**Figure 1:**
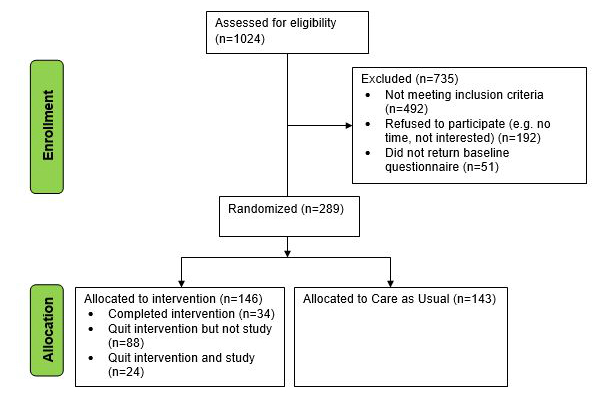
Flowchart of patient recruitment.

A detailed description of the patient recruitment for WEBCARE is displayed in Figure 1. A total of 1024 patients were approached for participation, 735 (71.78%) were excluded due to not meeting the inclusion criteria (n=492), refusing to participate (n=192), or not returning baseline measures (n=51). Eventually 289 patients were randomized to either the WC group (n=146) or the UC group (n=143).

Demographic, clinical, and psychological characteristics of patients who were randomized to the WC group are shown in Table 1. The mean age of the group was 58.23 (SD 9.87) and 120 (82.2%, 120/146) were male patients. In addition, 106 (72.6%, 106/146) had a higher educational level.

**Table 1:** Baseline demographic, clinical, and psychological characteristics of patients randomized to the WEBCARE treatment condition (WC; n=146).

Characteristic		WC group, mean (SD) or n (%)
**Demographics**		
	Age, mean (SD)	58.23 (9.87)
	Gender (male)	120 (82.2%)
	Employed	68 (46.6%)
	Has partner	124 (84.9%)
	High education	106 (72.6%)
**Clinical**		
	LVEF≤35^a^	87 (59.6%)
	QRS>120ms^b^, n=144	59 (41%)
	NYHA class III/IV^c^, n=122	20 (16.4%)
	Heart failure	78 (53.4%)
	CCI^d^, mean (SD)	1.60 (1.06)
	Beta-blockers	117 (80.1%)
	ACE-inhibitors	82 (56.2%)
	Diuretics	72 (49.3%)
	Psychotropic medication	13 (8.9%)
**Psychological**		
	Anxiety, mean (SD)	4.57 (5.02)
	Depression, mean (SD)	5.93 (5.11)
	Psychological treatment	8 (5.5%)
	Cardiac rehabilitation, n=145	20 (13.8%)
	Type D personality	24 (16.4%)
	Optimism, mean (SD)	11.23 (2.68)
	Pessimism, mean (SD)	5.73 (3.52)

^a^LVEF: left ventricular ejection fraction

^b^QRS-width: electrocardiogram Q,R, and S waves

^c^NYHA: New York Heart Association functional class

^d^CCI: Charlson Comorbidity Index

### Non-Participants

Patients who did not return the baseline questionnaires (n=51) and were excluded (not randomized) from current analyses did not differ systematically on demographic variables. However, significant differences on clinical variables were observed with patients who were not randomized, more often having a NYHA Class III/IV (*P*=.045), peripheral artery disease (*P*=.022), and using psychotropic medication (*P*≤.001) (anxiolytics, *P*=.004 and hypnotics, *P*=.010). Of the patients who refused to participate (n=192) but fulfilled the inclusion criteria, 60 (31.3%) signed consent and gave permission for medical record screening at the time of implantation. These patients were somewhat older [60.26 (SD 1.80) vs 58.16 (SD 10.30); *P*=.04], more likely to have a NYHA Class III/IV (34.1%, 14/41 vs 21.3%, 57/267; *P*=.013), to have experienced a previous myocardial infarction (76.6%, 36/47 vs 50.1%, 170/339; *P*=.001) or coronary artery bypass grafting (34.0%,16/47 vs 19.2%, 65/339; *P*=.019), to have peripheral artery disease (13.6%, 8/59 vs 5.3%, 18/339; *P*=.018), or to have a cardiac resynchronization therapy-defibrillator (CRT-D) (57.9%, 44/76 vs 25.9%, 87/336; *P*<.001) as compared to patients who signed informed consent for study participation (n=340).

### Adherence and Attrition

**Figure 2:**
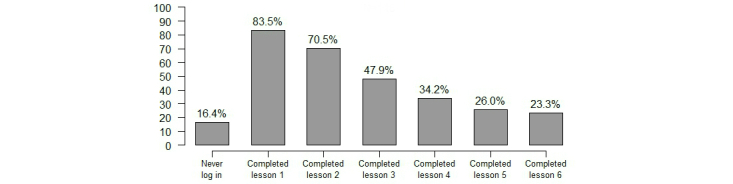
Adherence and attrition during the intervention (n=146).

Of the 146 randomized patients to the WC group, 34 (23.3%) completed the treatment and filled in the follow-up assessment (completers), 88 (60.0%) patients dropped out of the treatment but remained in the study and filled in the follow-up assessments (treatment dropouts), and 24 (16.4%) patients dropped out of the treatment and the study (dropouts). Focusing on the treatment, Figure 2 presents an overview of patients’ adherence to the intervention and shows that the number of patients completing the lessons diminishes over time. The first lesson was completed by 83.5% (122/146) of the patients randomized to the WC group (16.5%, 24/146 never logged in), while only 23.3% (34/146) completed the last lesson (and thus the whole treatment schedule).

### Differences in Baseline Characteristics and Psychological Profile

Completers and (treatment) dropouts did not systematically differ on any baseline demographic, clinical, or psychological measures (Table 2). Although not significant, dropouts tended to be more often employed, have a lower education level, and lower mean score on anxiety and depression as compared to the other two groups. Table 2 shows a detailed overview of the baseline demographic and clinical characteristics and psychological profile of the three groups.

**Table 2:** Baseline demographic, clinical, and psychological characteristics stratified by group.

Characteristic		Completers, n=34	Treatment dropout, n=88	Dropout, n=24	*P*
		mean (SD) or n (%)			
**Demographic**					
	Age, mean (SD)	57.91 (9.82)	58.84 (9.84)	56.42 (10.20)	.56
	Gender (male)	28 (82.4%)	74 (84.1%)	18 (75.0%)	.59
	Employed	15 (44.1%)	38 (43.2%)	15 (62.5%)	.23
	Has partner	30 (88.2%)	73 (83.0%)	21 (87.5%)	.71
	High education	26 (76.5%)	65 (74.7%)	15 (62.5%)	.43
**Clinical**					
	LVEF≤35^a^	21 (61.8%)	52 (59.1%)	14 (58.3%)	.96
	QRS≥120^b^	12 (36.4%)	37 (42.5%)	10 (41.7%)	.83
	NYHA III/IV^c^	4 (13.3%)	13 (20.0%)	3 (15.0%)	.69
	Heart failure	18 (52.9%)	46 (52.3%)	14 (58.3%)	.87
	CCI^d^, mean (SD)	1.88 (1.15)	1.56 (1.10)	1.38 (0.65)	.16
	Beta-blockers	28 (82.4%)	69 (78.4%)	20 (83.3%)	.81
	ACE-inhibitors	18 (52.9%)	52 (59.1%)	12 (50.0%)	.66
	Diuretics	16 (47.1%)	43 (48.9%)	13 (54.2%)	.86
	Psychotropics	1 (2.9%)	9 (10.2%)	3 (12.5%)	.36
**Psychological**					
	Anxiety, mean (SD)	5.46 (5.18)	4.21 (5.08)	4.63 (4.62)	.47
	Depression, mean (SD)	6.53 (4.40)	5.79 (5.38)	5.58 (5.17)	.73
	Type D	7 (20.6%)	14 (15.9%)	3 (12.5%)	.70
	Optimism, mean (SD)	11.12 (2.71)	11.41 (2.74)	10.75 (2.44)	.55
	Pessimism, mean (SD)	6.15 (4.16)	5.56 (3.40)	5.79 (2.99)	.71

^a^LVEF: left ventricular ejection fraction

^b^QRS-width: electrocardiogram Q,R, and S waves

^c^NYHA: New York Heart Association functional class

^d^CCI: Charlson Comorbidity Index

### Descriptive Data: Reasons for Dropout

**Figure 3:**
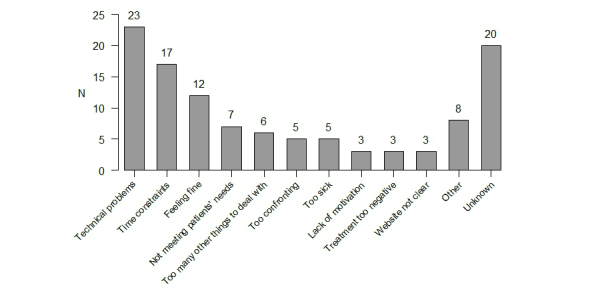
Reasons for dropout (n=112).

The reasons given by patients for not completing the treatment are displayed in Figure 3. Unfortunately, we were not able to reach all patients to learn about their reasons for dropout. Of the patients that we were able to reach, the majority (20.5%, 23/112) indicated that they faced technical problems (eg, the computer was not working, the website was not responding, problems with Internet connection): “My computer broke and I don’t use it that often, so I would have to buy another one just for the study; I didn’t wanted to do that” and “I got irritated because the website was not responding when I tried to log in, so I decided to quit”.

Time constraints were an issue in 15.2% (17/112) of patients (*“*I started working, so I don’t have time to do the online course”) and 10.7% (12/112) of patients were feeling fine and did not need additional support: “I had trouble thinking of any problems that I’m experiencing at this moment, as I don’t have any. I’m feeling fine”; and “I don’t have any problems. I’m happy that I have received the ICD. I feel reassured now”. Additional reasons were that the treatment did not apply to the patients’ needs (6.3%, 7/112): “I expected the course to be addressing only ICD specific problems…more technical problems. That’s why I decided to participate. I didn’t want to discuss psychological issues as I don’t have any”, and having already a lot to deal with (5.4%, 6/112): “I have just received an ICD and I’m dealing with depressive symptoms; I have other priorities at this moment”, and “I wanted to do the treatment but not within the 12 weeks. I wanted to start at a later time point because I had a lot to deal with immediately after the ICD implantation”.

Other reasons for dropout included treatment as being too confronting (4.5%, 5/112): “It was too personal, too confronting. I realized that I had more problems than I thought”, and feeling too sick (4.5%, 5/112): “It was too much. I had two surgeries in the past half year and I’m now on the waiting list for heart transplantation. I’m feeling sick all the time”. There were also patients who experienced the treatment as too negative: “I was feeling fine about my ICD, but when I started reading the content of the online course, I started feeling unhappy and therefore I decided to quit”, and “The homework assignments and questionnaires are too negatively worded, while you expect us to start thinking positive. I didn’t want to proceed as I didn’t wanted to start thinking negative about the ICD and how I’m feeling”.

## Discussion

### Principal Findings

This is the first behavioral randomized controlled trial to address the adherence and dropout issues of a Web-based intervention in the ICD population. Our findings show that 23.3% of patients randomized to the treatment arm completed the full treatment (six lessons), while 16.5% never logged on to the intervention. A gradual decline in adherence was observed with more patients dropping out as the lessons proceeded. The three groups (completers, treatment dropout, and dropout) did not differ systematically on any demographic or clinical baseline characteristics and their psychological profile. The top 3 reasons given for dropping out of the treatment were: technical issues with the computer/website, time constraints, and feeling fine/not needing additional support.

The findings of this study are generally in line with previous findings from the “Alles Onder Controle” Web-based intervention for individuals from the general population with increased levels of anxiety or depression who volunteered to undergo the intervention. Although generally higher percentages of completers were reported (38-55%), the rate of patients who never logged on was between 9% to 16% [25,26,35] and is in accordance with the 16.5%, which was observed in the current study. The higher number of completers in the Alles Onder Controle study might be attributed to these individuals being volunteers and thus highly motivated with respect to investing time and effort in improving their well-being. In addition, the latter patients scored high on anxiety and depressive symptoms, while patients in WEBCARE were included regardless of their anxiety/depression levels, as WEBCARE was set up as a prevention trial. However, there was a comparable gradual decline in adherence in the previous studies as in patients in the WEBCARE treatment arm [25,35].

In general, higher attrition rates are reported in open access Web-based trials without therapist guidance [36], while the number of completers is higher in closed trials where feedback is provided and reminders are sent [23,37]. Looking more specifically into cardiac patients (older adults with depression and comorbid cardiovascular disease), in their qualitative study on online treatment adherence, Donkin et al reported that time constraints, competing priorities, perception of limited worth of the intervention, and anxiety about spending time on the computer were important factors that contributed to an increased attrition [38], which are to some degree comparable to what we found in the current study. Generally, their results showed that adherence might increase if the benefits of participating in the treatment outweigh the costs of participation. In the current trial, the mean depression and anxiety scores were relatively low, patients were not distressed, and likely did not see a need for the treatment as they generally felt fine.

Studies of Web-based interventions have also shown that the duration of treatment [39] and timing [40] of the treatment may affect patients’ engagement. Also, patients’ perceived control over completing the course and coaching via phone vs email are found to be associated with higher completion rates [41]. In addition, printed delivery mode has shown to result in higher attrition as compared to a Web-based delivery mode [42]. These findings can partly explain why a higher attrition rate was observed in the current study. Current results show diminished participation after the third lesson. Perhaps, had the treatment been somewhat shorter, more patients would have completed all lessons. In addition, the treatment was offered immediately after ICD implantation. As indicated by some patients, at that time point they have a lot to deal with already and may for that reason decide not to participate or complete all modules.

### Limitations and Strengths

A number of limitations of this study must be acknowledged. First, current analyses are based on a relatively small sample and should be replicated in larger studies in the future. Second, results on reasons for dropout are based on descriptive data that were obtained via a telephone call to patients. A structured interview or validated questionnaire would perhaps provide more valid information. Third, unfortunately we were not able to reach all patients at 12 weeks by telephone; hence, current findings are based on patients who answered the phone and were willing to provide us with information regarding their reasons for dropout.

This study also has several strengths. To our knowledge, it is the first behavioral intervention trial in ICD patients to have used a Web-based approach and the first study in ICD patients overall to address issues of adherence and attrition. The information from WEBCARE adds to our knowledge about factors that may influence adherence in trials using a Web-based approach, which can be used when designing Web-based behavioral intervention trials for ICD patients in the future, in order to increase the number of patients enrolled in the study and their treatment adherence.

When offering a Web-based intervention to ICD patients, it seems of great importance to make the intervention as patient-tailored as possible. Not all patients have the same needs at the same time. Thus, giving them time and space to complete the lessons when needed is an important factor as is making it possible to select which lessons to complete (some patients are more interested in technical aspects of the ICD, which would give them more reassurance, while others prefer psychological support in dealing with this new situation). As a proportion of the ICD patients indicated that they were feeling fine and did not need any additional support, it might be more important to focus on patients who have higher distress levels post implant. These patients could be identified using brief and standardized questionnaires that are designed to assess psychological distress. Close monitoring of patients’ psychological needs is warranted as it has been associated with morbidity and mortality [11,12], and may help us to provide the right amount of care that is needed at the right time. Designing behavioral interventions with a collaborative care approach, where a period of “watchful waiting” is employed would perhaps be a way to go. With this approach, we would be able to offer support to patients who have “chronic” levels of distress or who develop distress at a later time point, as we have now learned that the “one size fits all” approach results in high dropout and low adherence. Incorporating a standard intervention in current health care models, which would be offered to all patients, might result in a great loss of resources as only a small proportion of patients would be willing to participate and complete the treatment (as shown in current data).

### Conclusions

In conclusion, as Web-based treatments are increasingly being implemented in clinical practice, knowing how to keep patients motivated and compliant with treatment becomes more important every day. Our findings indicate that more attention should be paid to the technical aspects of Web-based treatment and making it more user-friendly. In addition, to overcome the barrier of home computers not working as they should, future studies should examine whether a similar intervention could be delivered using smartphones or tablets in order to decrease dropout. Also, future studies should examine the relationship between adherence and outcomes, as the results to date are inconclusive [22]. Examining the appropriate duration and timing of the intervention is also of great importance, which to date remains unexplored in the ICD population. The provision of patient-tailored interventions at the time when the patient needs it is likely to increase treatment adherence and enhance the effectiveness of such interventions.

## Abbreviations

ANOVA: analysis of variance
CCI: Charlson Comorbidity Index
GAD: Generalized Anxiety Disorder scale
ICD: implantable cardioverter defibrillator
LVEF: left ventricular ejection fraction
NYHA class: New York Heart Association functional class
PHQ: Patient Health Questionnaire
QRS: electrocardiogram Q, R, and S waves
RCT: randomized controlled trial
UC: usual care (group)
WC: WEBCARE (group)
WEBCARE: WEB-based distress management program for implantable CARdioverter dEfibrillator patients

## Multimedia Appendix 1

CONSORT-EHEALTH checklist V1.6.2 [43].

## References

[1] Ezekowitz JA, Armstrong PW, McAlister FA (2003). Implantable cardioverter defibrillators in primary and secondary prevention: a systematic review of randomized, controlled trials. Ann Intern Med.

[2] Zipes DP, Camm AJ, Borggrefe M, Buxton AE, Chaitman B, Fromer M, Gregoratos G, Klein G, Moss AJ, Myerburg RJ, Priori SG, Quinones MA, Roden DM, Silka MJ, Tracy C, Smith SC, Jacobs AK, Adams CD, Antman EM, Anderson JL, Hunt SA, Halperin JL, Nishimura R, Ornato JP, Page RL, Riegel B, Priori SG, Blanc JJ, Budaj A, Camm AJ, Dean V, Deckers JW, Despres C, Dickstein K, Lekakis J, McGregor K, Metra M, Morais J, Osterspey A, Tamargo JL, Zamorano JL, European Heart Rhythm Association, Heart Rhythm Society, American College of Cardiology, American Heart Association Task Force, European Society of Cardiology Committee for Practice Guidelines (2006). ACC/AHA/ESC 2006 guidelines for management of patients with ventricular arrhythmias and the prevention of sudden cardiac death: a report of the American College of Cardiology/American Heart Association Task Force and the European Society of Cardiology Committee for Practice Guidelines (Writing Committee to Develop Guidelines for Management of Patients With Ventricular Arrhythmias and the Prevention of Sudden Cardiac Death). J Am Coll Cardiol.

[3] Sears SF, Kirian K (2010). Shock and patient-centered outcomes research: is an ICD shock still a critical event?. Pacing Clin Electrophysiol.

[4] Pedersen SS, Van Den Broek KC, Van Den Berg M, Theuns DA (2010). Shock as a determinant of poor patient-centered outcomes in implantable cardioverter defibrillator patients: is there more to it than meets the eye?. Pacing Clin Electrophysiol.

[5] Magyar-Russell G, Thombs BD, Cai JX, Baveja T, Kuhl EA, Singh PP, Montenegro Braga Barroso M, Arthurs E, Roseman M, Amin N, Marine JE, Ziegelstein RC (2011). The prevalence of anxiety and depression in adults with implantable cardioverter defibrillators: a systematic review. J Psychosom Res.

[6] Habibović M, van den Broek KC, Alings M, Van der Voort PH, Denollet J (2012). Posttraumatic stress 18 months following cardioverter defibrillator implantation: shocks, anxiety, and personality. Health Psychol.

[7] Habibović M, Versteeg H, Pelle AJ, Theuns DA, Jordaens L, Pedersen SS (2013). Poor health status and distress in cardiac patients: the role of device therapy vs. underlying heart disease. Europace.

[8] Pedersen SS, Hoogwegt MT, Jordaens L, Theuns DA (2011). Relation of symptomatic heart failure and psychological status to persistent depression in patients with implantable cardioverter-defibrillator. Am J Cardiol.

[9] Pedersen SS, Hoogwegt MT, Jordaens L, Theuns DA (2013). Pre implantation psychological functioning preserved in majority of implantable cardioverter defibrillator patients 12 months post implantation. Int J Cardiol.

[10] Braunschweig F, Boriani G, Bauer A, Hatala R, Herrmann-Lingen C, Kautzner J, Pedersen SS, Pehrson S, Ricci R, Schalij MJ (2010). Management of patients receiving implantable cardiac defibrillator shocks: recommendations for acute and long-term patient management. Europace.

[11] Habibović M, Pedersen SS, van den Broek KC, Theuns DA, Jordaens L, van der Voort PH, Alings M, Denollet J (2013). Anxiety and risk of ventricular arrhythmias or mortality in patients with an implantable cardioverter defibrillator. Psychosom Med.

[12] Pedersen SS, Brouwers C, Versteeg H (2012). Psychological vulnerability, ventricular tachyarrhythmias and mortality in implantable cardioverter defibrillator patients: is there a link?. Expert Rev Med Devices.

[13] Hoogwegt MT, Kupper N, Theuns DA, Zijlstra WP, Jordaens L, Pedersen SS (2012). Undertreatment of anxiety and depression in patients with an implantable cardioverter-defibrillator: impact on health status. Health Psychol.

[14] Habibović M, Burg MM, Pedersen SS (2013). Behavioral interventions in patients with an implantable cardioverter defibrillator: lessons learned and where to go from here?. Pacing Clin Electrophysiol.

[15] Pedersen SS, van den Broek KC, Sears SF (2007). Psychological intervention following implantation of an implantable defibrillator: a review and future recommendations. Pacing Clin Electrophysiol.

[16] Salmoirago-Blotcher E, Ockene IS (2009). Methodological limitations of psychosocial interventions in patients with an implantable cardioverter-defibrillator (ICD): A systematic review. BMC Cardiovasc Disord.

[17] Dickerson SS, Flaig DM, Kennedy MC (2000). Therapeutic connection: help seeking on the Internet for persons with implantable cardioverter defibrillators. Heart Lung.

[18] Andrews G, Cuijpers P, Craske MG, McEvoy P, Titov N (2010). Computer therapy for the anxiety and depressive disorders is effective, acceptable and practical health care: a meta-analysis. PLoS One.

[19] Cuijpers P, van Straten A, Andersson G (2008). Internet-administered cognitive behavior therapy for health problems: A systematic review. J Behav Med.

[20] Taylor CB, Jobson KO, Winzelberg A, Abascal L (2002). The use of the Internet to provide evidence-based integrated treatment programs for mental health. Psychiat Ann.

[21] Proudfoot JG (2004). Computer-based treatment for anxiety and depression: is it feasible? Is it effective?. Neurosci Biobehav Rev.

[22] Donkin L, Christensen H, Naismith SL, Neal B, Hickie IB, Glozier N (2011). A systematic review of the impact of adherence on the effectiveness of e-therapies. J Med Internet Res.

[23] Eysenbach G (2005). The law of attrition. J Med Internet Res.

[24] Pedersen SS, Spek V, Theuns DA, Alings M, van der Voort P, Jordaens L, Cuijpers P, Denollet J, van den Broek KC (2009). Rationale and design of WEBCARE: a randomized, controlled, web-based behavioral intervention trial in cardioverter-defibrillator patients to reduce anxiety and device concerns and enhance quality of life. Trials.

[25] Warmerdam L, van Straten A, Twisk J, Riper H, Cuijpers P (2008). Internet-based treatment for adults with depressive symptoms: randomized controlled trial. J Med Internet Res.

[26] van Straten A, Cuijpers P, Smits N (2008). Effectiveness of a web-based self-help intervention for symptoms of depression, anxiety, and stress: randomized controlled trial. J Med Internet Res.

[27] Charlson ME, Pompei P, Ales KL, MacKenzie CR (1987). A new method of classifying prognostic comorbidity in longitudinal studies: development and validation. J Chronic Dis.

[28] Spitzer RL, Kroenke K, Williams JB, Löwe B (2006). A brief measure for assessing generalized anxiety disorder: the GAD-7. Arch Intern Med.

[29] Kroenke K, Spitzer RL, Williams JB (2001). The PHQ-9: validity of a brief depression severity measure. J Gen Intern Med.

[30] McManus D, Pipkin SS, Whooley MA (2005). Screening for depression in patients with coronary heart disease (data from the Heart and Soul Study). Am J Cardiol.

[31] Denollet J, Sys SU, Stroobant N, Rombouts H, Gillebert TC, Brutsaert DL (1996). Personality as independent predictor of long-term mortality in patients with coronary heart disease. Lancet.

[32] Emons WH, Meijer RR, Denollet J (2007). Negative affectivity and social inhibition in cardiovascular disease: evaluating type-D personality and its assessment using item response theory. J Psychosom Res.

[33] Denollet J (2005). DS14: standard assessment of negative affectivity, social inhibition, and Type D personality. Psychosom Med.

[34] Scheier MF, Carver CS, Bridges MW (1994). Distinguishing optimism from neuroticism (and trait anxiety, self-mastery, and self-esteem): a reevaluation of the Life Orientation Test. J Pers Soc Psychol.

[35] Warmerdam L, van Straten A, Jongsma J, Twisk J, Cuijpers P (2010). Online cognitive behavioral therapy and problem-solving therapy for depressive symptoms: Exploring mechanisms of change. J Behav Ther Exp Psychiatry.

[36] Christensen H, Griffiths KM, Korten AE, Brittliffe K, Groves C (2004). A comparison of changes in anxiety and depression symptoms of spontaneous users and trial participants of a cognitive behavior therapy website. J Med Internet Res.

[37] Wanner M, Martin-Diener E, Bauer G, Braun-Fahrländer C, Martin BW (2010). Comparison of trial participants and open access users of a web-based physical activity intervention regarding adherence, attrition, and repeated participation. J Med Internet Res.

[38] Donkin L, Glozier N (2012). Motivators and motivations to persist with online psychological interventions: a qualitative study of treatment completers. J Med Internet Res.

[39] Christensen H, Griffiths KM, Mackinnon AJ, Brittliffe K (2006). Online randomized controlled trial of brief and full cognitive behaviour therapy for depression. Psychol Med.

[40] Strecher VJ, McClure J, Alexander G, Chakraborty B, Nair V, Konkel J, Greene S, Couper M, Carlier C, Wiese C, Little R, Pomerleau C, Pomerleau O (2008). The role of engagement in a tailored web-based smoking cessation program: randomized controlled trial. J Med Internet Res.

[41] Wojtowicz M, Day V, McGrath PJ (2013). Predictors of participant retention in a guided online self-help program for university students: prospective cohort study. J Med Internet Res.

[42] Peels DA, Bolman C, Golsteijn RH, De Vries H, Mudde AN, van Stralen MM, Lechner L (2012). Differences in reach and attrition between Web-based and print-delivered tailored interventions among adults over 50 years of age: clustered randomized trial. J Med Internet Res.

[43] Eysenbach G, CONSORT-EHEALTH Group (2011). CONSORT-EHEALTH: improving and standardizing evaluation reports of Web-based and mobile health interventions. J Med Internet Res.

