# Supplementary material for: Attrition and Adherence in a Web-Based Distress Management Program for Implantable Cardioverter Defibrillator Patients (WEBCARE): Randomized Controlled Trial
Source: J Med Internet Res. 2014 Feb 28;16(2):e52. doi: 10.2196/jmir.2809 (PMC3961745; doi:10.2196/jmir.2809)
Supplement: Supplementary file 1 [file jmir_v16i2e52_app1.pdf]

CONSORT-EHEALTH Checklist V1.6.2 Report

(based on CONSORT-EHEALTH V1.6), available at [http://tinyurl.com/consort-ehealth-v1-6].

2809

Date completed

2/14/2014 5:49:27

by

Susanne Pedersen

Attrition and adherence in the web-based distress management program for implantable cardioverter defibrillator patients (WEBCARE) trial

TITLE

1a-i) Identify the mode of delivery in the title

"web-based"

1a-ii) Non-web-based components or important co-interventions in title

"patients received a relaxation training CD which they were allowed to use throughout the intervention". This is stated in the 'intervention' section of the manuscript. As this was not a web-based part of the intervention we did not mention it in the title.

1a-iii) Primary condition or target group in the title

"implantable cardioverter defibrillator patients"

ABSTRACT

1b-i) Key features/functionalities/components of the intervention and comparator in the METHODS section of the ABSTRACT

The primary focus of the current study is to describe attrition and adherence within the WEBCARE trial. A detailed description of the intervention is provided in the manuscript but was not included in the abstract.

1b-ii) Level of human involvement in the METHODS section of the ABSTRACT

In the abstract we did not further described the intervention components or the level of human involvement as we strongly focused on the attrition and adherence in this study.

1b-iii) Open vs. closed, web-based (self-assessment) vs. face-to-face assessments in the METHODS section of the ABSTRACT

"Consecutive first-time ICD patients from 6 Dutch referral hospitals were approached for participation."

1b-iv) RESULTS section in abstract must contain use data

"The treatment arm of WEBCARE contained 146 patients. Of these 146, 34 (23.3%) completed the treatment, 88 (60.3%) dropped out of treatment but completed follow-up, and 24 (16.4%) dropped out of treatment and study."

1b-v) CONCLUSIONS/DISCUSSION in abstract for negative trials

"Current findings underline the importance of focusing on adherence and dropout, as this remains a significant problem in behavioral web-based trials."

INTRODUCTION

2a-i) Problem and the type of system/solution

"Results of previous trials have been promising with respect to reducing distress, but the majority of these trials had a high drop-out rate jeopardizing the external validity of these studies. In order to make psychological treatment for ICD patients more patient tailored, which may reduce dropout, the use of online web-based interventions has been advocated."

2a-ii) Scientific background, rationale: What is known about the (type of) system

"To date the reasons for dropout are not well understood and deserve attention in their own right, in order to increase the success and applicability of results of future web-based intervention trials."

METHODS

3a) CONSORT: Description of trial design (such as parallel, factorial) including allocation ratio

"Specific objectives will be to: (1) examine whether (a) completers, (b) patients who dropped-out of treatment but who remained in de study (filled in follow-up questionnaires), and (c) patients who dropped-out of the treatment and the study differ systematically on baseline demographic, clinical, and psychological characteristics; and (2) present descriptive data on the reasons of patients for dropping out."

3b) CONSORT: Important changes to methods after trial commencement (such as eligibility criteria), with reasons

We did not make any changes to the methods.

3b-i) Bug fixes, Downtimes, Content Changes

No changes were made.

4a) CONSORT: Eligibility criteria for participants

"Patients were eligible for participation if they fulfilled the following inclusion criteria and none of the exclusion criteria: Inclusion criteria: First-time ICD implant; age 18-75 years; proficient in the Dutch language; and internet access and a sufficient level of internet skills. Exclusion criteria: Life expectancy less than 1 year; history of psychiatric illness other than affective/anxiety disorders; or on the waiting list for heart transplantation."

4a-i) Computer / Internet literacy

"internet access and a sufficient level of internet skills"

4a-ii) Open vs. closed, web-based vs. face-to-face assessments:

"Patients were approached by the ICD nurse or ICD technician prior to, or briefly after ICD implantation."

"The online course for ICD patients is a 12-week intervention of 6 online lessons"

"From lesson two patients received homework assignments and were provided with therapist feedback (feedback was provided by master-level educated psychologists and was intended as minimal guidance to help patients get through the lessons – encouraging patients to continue with the lessons and giving guidance in how to address their problems according to the problem solving theory). In addition, patients received a relaxation training CD which they were allowed to use throughout the intervention."

4a-iii) Information giving during recruitment

"They were informed both orally and in writing about the study."

4b) CONSORT: Settings and locations where the data were collected

"Prior to discharge from the hospital, consented patients were provided with the first set of questionnaires (baseline) and their medical records were accessed for information on their demographic and clinical variables."

4b-i) Report if outcomes were (self-)assessed through online questionnaires

Patients did not fill in the questionnaires online.

"After completing the questionnaires, patients returned them in a self-addressed and pre-stamped envelope to Tilburg University that served as the core lab for WEBCARE."

4b-ii) Report how institutional affiliations are displayed

5) CONSORT: Describe the interventions for each group with sufficient details to allow replication, including how and when they were actually administered
5-i) Mention names, credential, affiliations of the developers, sponsors, and owners
"The intervention was based on the previously developed online treatment 'Alles Onder Controle' (Everything Under Control) and was for the purpose of the WEBCARE trial adapted for ICD patients."
5-ii) Describe the history/development process
The existing 'Everything Under Control" intervention was adapted for ICD patients. During the adaptation phase an advisor from the ICD patient organization was involved in the proces (also an ICD patient). Important imput was provided regarding the wording of the teksts and problems that are encounterd within this patient population.
5-iii) Revisions and updating
Not applicable
5-iv) Quality assurance methods
Not applicable
5-v) Ensure replicability by publishing the source code, and/or providing screenshots/screen-capture video, and/or providing flowcharts of the algorithms used
The intervention has previously been developed and published by others.
5-vi) Digital preservation
Not applicable
5-vii) Access
"The WEBCARE (WC) group, receiving questionnaires at baseline, 3 months, 6 months, and 12 months by mail, and getting access to the internet intervention for a time period of 12 weeks to complete six modules online."
5-viii) Mode of delivery, features/functionalities/components of the intervention and comparator, and the theoretical framework
"The intervention was based on the previously developed online treatment 'Alles Onder Controle' (Everything Under Control)[25] and was for the purpose of the WEBCARE trial adapted for ICD patients. The 'Alles Onder Controle' treatment was developed for the healthy depressed population and has proven to be effective in reducing distress [25, 26]. The online course for ICD patients is a 12-week intervention of 6 online lessons addressing distress based on the cognitive behavioral model (problem solving treatment). Lesson one focused on psycho-education with respect to living with an ICD (e.g. what are 'normal' adaptation problems post ICD implantation). From lesson two patients received homework assignments and were provided with therapist feedback (feedback was provided by master-level educated psychologists and was intended as minimal guidance to help patients get through the lessons – encouraging patients to continue with the lessons and giving guidance in how to address their problems according to the problem solving theory). In addition, patients received a relaxation training CD which they were allowed to use throughout the intervention.
Patients were allowed to work at their own time and pace, however, if a lesson was not finished within two weeks a reminder e-mail was sent, with up to three reminders per lesson. Patients could only proceed to the next lesson when the previous one was finished and the homework assignment was sent to the therapist. If patients did not log in within the first two weeks, a reminder e-mail was sent. Twelve weeks after receiving the login information, patients' accounts were automatically closed."
5-ix) Describe use parameters
"Patients were allowed to work at their own time and pace, however, if a lesson was not finished within two weeks a reminder e-mail was sent, with up to three reminders per lesson. Patients could only proceed to the next lesson when the previous one was finished and the homework assignment was sent to the therapist. If patients did not log in within the first two weeks, a reminder e-mail was sent. Twelve weeks after receiving the login information, patients' accounts were automatically closed."
5-x) Clarify the level of human involvement
"From lesson two patients received homework assignments and were provided with therapist feedback (feedback was provided by master-level educated psychologists and was intended as minimal guidance to help patients get through the lessons – encouraging patients to continue with the lessons and giving guidance in how to address their problems according to the problem solving theory)."
5-xi) Report any prompts/reminders used
" ..if a lesson was not finished within two weeks a reminder e-mail was sent, with up to three reminders per lesson. Patients could only proceed to the next lesson when the previous one was finished and the homework assignment was sent to the therapist. If patients did not log in within the first two weeks, a reminder e-mail was sent."
5-xii) Describe any co-interventions (incl. training/support)
"In addition, patients received a relaxation training CD which they were allowed to use throughout the intervention."
6a) CONSORT: Completely defined pre-specified primary and secondary outcome measures, including how and when they were assessed
"Specific objectives will be to: (1) examine whether (a) completers, (b) patients who dropped-out of treatment but who remained in de study (filled in follow-up questionnaires), and (c) patients who dropped-out of the treatment and the study differ systematically on baseline demographic, clinical, and psychological characteristics; and (2) present descriptive data on the reasons of patients for dropping out."

"Prior to discharge from the hospital, consented patients were provided with the first set of questionnaires (baseline) and their medical records were accessed for information on their demographic and clinical variables. After completing the questionnaires, patients returned them in a self-addressed and pre-stamped envelope to Tilburg University that served as the core lab for WEBCARE."

"Patients who signed the informed consent form but who decided to prematurely quit the intervention and/or the study were contacted by telephone 12 weeks after randomization and asked why they had decided to quit. This time interval was chosen in order not to interfere with possible intervention effects (patients were allowed to work at their own pace, some chose to finish the intervention within the first two weeks, while others decided to do the 6 lessons within the last two weeks. For that reason it was clear at 12 weeks who quit / finished the intervention). Hence, patients were contacted at the time that they should have received their 3 months follow-up and finished the 6-module online course."
6a-i) Online questionnaires: describe if they were validated for online use and apply CHERRIES items to describe how the questionnaires were designed/deployed
Not applicable - no online questionnaires were used.
6a-ii) Describe whether and how "use" (including intensity of use/dosage) was defined/measured/monitored
Not applicable
6a-iii) Describe whether, how, and when qualitative feedback from participants was obtained

"Patients who signed the informed consent form but who decided to prematurely quit the intervention and/or the study were contacted by telephone 12 weeks after randomization and asked why they had decided to quit. This time interval was chosen in order not to interfere with possible intervention effects (patients were allowed to work at their own pace, some chose to finish the intervention within the first two weeks, while others decided to do the 6 lessons within the last two weeks. For that reason it was clear at 12 weeks who quit / finished the intervention). Hence, patients were contacted at the time that they should have received their 3 months follow-up and finished the 6-module online course."

6b) CONSORT: Any changes to trial outcomes after the trial commenced, with reasons

No changes to outcomes were made.

7a) CONSORT: How sample size was determined

7a-i) Describe whether and how expected attrition was taken into account when calculating the sample size

Not applicable for current study

7b) CONSORT: When applicable, explanation of any interim analyses and stopping guidelines

Not applicable

8a) CONSORT: Method used to generate the random allocation sequence

"Patients were randomized using block randomization by computer, randomizing 20 patients per hospital, at each time point..Randomization lists were generated by an independent, blinded statistician and sealed by a research assistant. "

8b) CONSORT: Type of randomisation; details of any restriction (such as blocking and block size)

"Patients were randomized using block randomization by computer, randomizing 20 patients per hospital, at each time point."

9) CONSORT: Mechanism used to implement the random allocation sequence (such as sequentially numbered containers), describing any steps taken to conceal the sequence until interventions were assigned

Upon returning the baseline questionnaires, and prior to opening the envelope, patiets were randomized on a 1:1 basis by drawing a sealed envelope for each patient containing the condition.

10) CONSORT: Who generated the random allocation sequence, who enrolled participants, and who assigned participants to interventions

"Randomization lists were generated by an independent, blinded statistician and sealed by a research assistant. "

"Patients were approached by the ICD nurse or ICD technician prior to, or briefly after ICD implantation."

The research assistant assigned the participants to the intervention by drawing a sealed envelope for each patient (containing the intervention).

11a) CONSORT: Blinding - If done, who was blinded after assignment to interventions (for example, participants, care providers, those assessing outcomes) and how

11a-i) Specify who was blinded, and who wasn't

Blinding of participants, coaches, or care providers was not possible. Although the care providers were not informed about the study condition of the participating patients, there is always the possibility that the patient might have informed the health care provider about it.

11a-ii) Discuss e.g., whether participants knew which intervention was the "intervention of interest" and which one was the "comparator"

11b) CONSORT: If relevant, description of the similarity of interventions

Not applicable

12a) CONSORT: Statistical methods used to compare groups for primary and secondary outcomes

"Continuous variables were compared using the Student's t-test, while discrete variables were compared using the X2 test. Data are represented as percentages for nominal variables and mean (SDs) for continuous variables. To compare groups on psychological variables ANOVAs were performed. If group differences were observed, the Tukey-Kramer post-hoc test for unequal group sizes was used to identify which groups differed significantly. Descriptive data were coded and analyzed using 'frequencies'. A P<.05 indicated statistical significance. All tests were two-tailed. Data were analyzed using SPSS Statistics 19.0 for Windows."

12a-i) Imputation techniques to deal with attrition / missing values

Not applicable for current analyses.

12b) CONSORT: Methods for additional analyses, such as subgroup analyses and adjusted analyses

Not applicable for current study.

RESULTS

13a) CONSORT: For each group, the numbers of participants who were randomly assigned, received intended treatment, and were analysed for the primary outcome

"A total of 1024 patients were approached for participation, 735 (72%) were excluded due to not meeting the inclusion criteria (n=492), refusing to participate (n=192), or not returning baseline measures (n=51). Eventually 289 patients were randomized to either the WC group (n=146) or the UC group (n=143)."

"Of the 146 randomized patients to the WC group, 34 (23.3%) completed the treatment and filled in the follow-up assessment (completers), 88 (60.0%) patients dropped out of the treatment but remained in the study and filled in the follow-up assessments (treatment dropouts), and 24 (16.4%) patients dropped out of the treatment and the study (dropouts)."

13b) CONSORT: For each group, losses and exclusions after randomisation, together with reasons

See Figure 1: Flowchart of patient recruitment. Data presented in the CONSORT flow diagram.

13b-i) Attrition diagram

Data presented in CONSORT flow diagram as described in results.

"Of the 146 randomized patients to the WC group, 34 (23.3%) completed the treatment and filled in the follow-up assessment (completers), 88 (60.0%) patients dropped out of the treatment but remained in the study and filled in the follow-up assessments (treatment dropouts), and 24 (16.4%) patients dropped out of the treatment and the study (dropouts). Focussing on the treatment, Figure 2 presents an overview of patients' adherence to the intervention and shows that the number of patients completing the lessons diminishes over time. The first lesson was completed by 83.5% of the patients randomized to the WC group (16.5% never logged in), while only 23.3% completed the last lesson (and thus the whole treatment schedule)."

14a) CONSORT: Dates defining the periods of recruitment and follow-up

".....for study participation between April 2010 and March 2013."

14a-i) Indicate if critical "secular events" fell into the study period

Not applicable

14b) CONSORT: Why the trial ended or was stopped (early)

The trial did not stop earlier than planned.

15) CONSORT: A table showing baseline demographic and clinical characteristics for each group

"Table 1: Baseline demographic, clinical, and psychological characteristics of patients randomized to the WEBCARE treatment condition"

"Table 2: Baseline demographic, clinical, and psychological characteristics stratified by group"

15-i) Report demographics associated with digital divide issues

In the current trial only patient with sufficient internet literacy were included. However, the level of literacy is not known. Therefore we were not able to indicate possible demographic associates with internet literacy.

16a) CONSORT: For each group, number of participants (denominator) included in each analysis and whether the analysis was by original assigned groups

16-i) Report multiple "denominators" and provide definitions

Throughout the manuscript N was provided for all presented data.

16-ii) Primary analysis should be intent-to-treat

Not applicable for current study.

17a) CONSORT: For each primary and secondary outcome, results for each group, and the estimated effect size and its precision (such as 95% confidence interval)

Not applicable for current study.

17a-i) Presentation of process outcomes such as metrics of use and intensity of use

Not applicable for current study.

17b) CONSORT: For binary outcomes, presentation of both absolute and relative effect sizes is recommended

Not applicable.

18) CONSORT: Results of any other analyses performed, including subgroup analyses and adjusted analyses, distinguishing pre-specified from exploratory

Not applicable for current study.

18-i) Subgroup analysis of comparing only users

19) CONSORT: All important harms or unintended effects in each group

Not applicable for current study.

19-i) Include privacy breaches, technical problems

19-ii) Include qualitative feedback from participants or observations from staff/researchers

DISCUSSION

20) CONSORT: Trial limitations, addressing sources of potential bias, imprecision, multiplicity of analyses

20-i) Typical limitations in ehealth trials

"A number of limitations of this study must be acknowledged. First, current analyses are based on a relatively small sample and should be replicated in larger studies in the future. Second, results on reasons for drop-out are based on descriptive data which were obtained via a telephone call to patients. A structured interview or validated questionnaire would perhaps provide more valid information. Third, unfortunately we were not able to reach all patients at 12-weeks by telephone, hence current findings are based on patients who answered the phone and were willing to provide us with information regarding their reasons for drop-out."

21) CONSORT: Generalisability (external validity, applicability) of the trial findings

21-i) Generalizability to other populations

21-ii) Discuss if there were elements in the RCT that would be different in a routine application setting

22) CONSORT: Interpretation consistent with results, balancing benefits and harms, and considering other relevant evidence

22-i) Restate study questions and summarize the answers suggested by the data, starting with primary outcomes and process outcomes (use)

"Our findings show that 23.3% of patients randomized to the treatment arm completed the full treatment (six lessons), while 16.5% never logged on the intervention. A gradual decline in adherence was observed with more patients dropping out as the lessons proceeded. The three groups (completers; treatment dropout; dropout) did not differ systematically on any demographic or clinical baseline characteristics and their psychological profile. The top 3 reasons given for dropping out of the treatment were: technical issues with the computer / website, time constraints, and feeling fine not needing additional support."

22-ii) Highlight unanswered new questions, suggest future research

"Our findings indicate that more attention should be paid to the technical aspects of the online treatment and making it more user friendly. In addition, to overcome the barrier of home computers not working as they should, future studies should examine whether a similar intervention could be delivered using smart phones or tablets in order to decrease drop-out. Also, future studies should examine the relationship between adherence and outcomes as the results to date are inconclusive [22]. Examining the appropriate duration and timing of the intervention is also of great importance, which to date remains unexplored in the ICD population. The provision of patient tailored interventions at the time when the patient needs it is likely to increase treatment adherence and enhance the effectiveness of such interventions."

Other information

23) CONSORT: Registration number and name of trial registry

"Trial registration: http://www.ClinicalTrials.gov. Identifier: NCT00895700"

24) CONSORT: Where the full trial protocol can be accessed, if available

"The trial design paper has been published previously [24]."

25) CONSORT: Sources of funding and other support (such as supply of drugs), role of funders

"This work was in part supported with grant no. 300020002 (with support of the Dutch Heart foundation) and a VIDI grant (91710393) from the Netherlands Organization for Health Research and Development (ZonMW), The Hague, The Netherlands, to Dr. Susanne S. Pedersen."

X26-i) Comment on ethics committee approval

"The study was approved by the Medical Ethics Committee of all participating centres and was conducted in accordance with the Helsinki declaration. All patients provided written informed consent."

x26-ii) Outline informed consent procedures

"Patients were approached by the ICD nurse or ICD technician prior to, or briefly after ICD implantation. They were informed both orally and in writing about the study. If the patient met the inclusion criteria and was willing to participate, informed consent was signed."

X26-iii) Safety and security procedures

Not applicable for current study.

X27-i) State the relation of the study team towards the system being evaluated
"CONFLICST OF INTEREST

None."
